# *Sa*UspA, the Universal Stress Protein of *Sulfolobus acidocaldarius* Stimulates the Activity of the PP2A Phosphatase and Is Involved in Growth at High Salinity

**DOI:** 10.3389/fmicb.2020.598821

**Published:** 2020-11-13

**Authors:** Xing Ye, Chris van der Does, Sonja-Verena Albers

**Affiliations:** Molecular Biology of Archaea, Faculty of Biology, Institute of Biology II, University of Freiburg, Freiburg, Germany

**Keywords:** Sulfolobus acidocaldarius, protein phosphatase, universal stress protein, ATP binding, archaellin, high salinity stress, archaea

## Abstract

In *Sulfolobus acidocaldarius*, the protein phosphatase PP2A plays important regulatory roles in many cellular processes, including cell growth, cell shape and synthesis of the archaellum. A conserved prokaryotic protein, designated as *Sa*UspA, was identified as an interaction partner of the phosphatase PP2A. *Sa*UspA belongs to the universal stress protein (USP) superfamily, members of which are found in bacteria, archaea, plants and invertebrates. Biochemical analysis showed that *Sa*UspA is a homodimeric ATP-binding protein, which also *in vitro* binds to PP2A. *Sa*UspA did not hydrolyze ATP, but stimulated the phosphatase activity of PP2A and might in this manner affect many other processes. Interestingly, binding of ATP further enhanced *Sa*UspA’s interaction with PP2A. In contrast to bacterial *usp* genes, environmental stress conditions including stationary phase, starvation stress, high salinity stress and UV stress did not stimulate expression of *saUspA*. Deletion of *saUspA* led to premature production of the archaellin FlaB in *S. acidocaldarius* although motility was not affected. The Δ*saUspA* mutant showed a significant growth defect under high salinity stress and complementation of ATP-binding deficient mutant *Sa*UspA^G97A^ failed to restore this growth defect. Compared with the wild type strain, its growth or survival was not affected under heavy metal stress and UV stress. To date, this is the first study in which the physiological role of USP homologs in archaea have been reported.

## Introduction

The universal stress protein (USP) superfamily is highly conserved in bacteria, archaea, plants and other eukaryotes (Vollmer and Bark, 2018). Expression of members of this family is triggered by a multitude of different environmental stress conditions, which suggests an important role of these proteins in general stress responses (Kvint et al., 2003; Vollmer and Bark, 2018). In the past three decades, significant effort has been made to explore the physiological functions of members of the USP superfamily in bacteria and plants. In bacteria, USP proteins were found to be involved in cell growth and survival under stress conditions, motility, biofilm formation, and virulence of pathogen to host (Kvint et al., 2003; Nachin et al., 2005; O’Connor and McClean, 2017; Vollmer and Bark, 2018). In plants, these proteins were critical for the tolerance against abiotic stresses including drought, osmotic stress and anoxia (Gonzali et al., 2015; Gutiérrez-beltrán et al., 2017; Vollmer and Bark, 2018).

UspA was originally discovered in *Escherichia coli* and was the founding member of the USP superfamily (Nystrdm and Neidhardt, 1992). Later, five paralogs of UspA were identified in *E. coli*, including UspC, UspD, UspE, UspF, and UspG (Gustavsson et al., 2002). A number of environmental stress conditions, such as nutrient starvation (carbon source, phosphate, sulfur and nitrogen) and osmotic stress, heat shock, UV exposure, and exposure to high salinity or heavy metals induced their expression (Kvint et al., 2003). With the increasing amount of sequenced genomes, more and more USP family members were found in different kinds of organisms such as bacteria, archaea, fungi, plants and invertebrates (Vollmer and Bark, 2018). USP superfamily members are divided into two major groups: non-ATP-binding and ATP-binding members (Kvint et al., 2003; Vollmer and Bark, 2018). UspA from *E. coli* and *Haemophilus influenzae* are the representative members of the first group, and the second group is represented by MJ0577 from *Methanocaldococcus jannaschii* (Zarembinski et al., 1998; Sousa and Mckay, 2001). Notably, ATP-binding is important for the function of the ATP-binding USP proteins. In *Mycobacterium tuberculosis*, ATP-binding deficient mutants of USP homologs Rv2623 and Rv2624c failed to regulate cell growth and pathogenicity (Drumm et al., 2009). Homodimer formation of *E. coli* USP proteins and heterodimer formation of different USP proteins with each other were shown to be responsible for the functional diversity in response to different stresses (Vollmer and Bark, 2018). In *E. coli*, UspA, UspC, and UspD formed homo- and hetero-dimers, and UspF and UspG can form three types of dimers (Nachin et al., 2008). In addition, some members of USP superfamily were phosphorylated *in vitro*/*vivo*. Phosphorylation of UspA in *E. coli* occurred specifically at serine/threonine residues *in vivo*, which depended on the TypA tyrosine phosphoprotein (Freestone et al., 1997). With cell lysates, the autophosphorylation of UspA occurs *in vitro*. UspG also showed autophosphorylation activity *in vitro* (Weber and Jung, 2006). In *M. tuberculosis*, threonine-237 of Rv2623 was found to be phosphorylated, and mutation to alanine decreased its interaction with a putative ATP binding cassette transporter Rv1747 and abolished its regulatory function in cell growth (Glass et al., 2017).

As described above, USP proteins are rather well studied in bacteria and plants. Although the structures of USP proteins from *M. jannaschii* and *Archaeoglobus fulgidus* have been determined (Zarembinski et al., 1998; Tkaczuk et al., 2013), the physiological roles of USP proteins have not been addressed in archaea yet.

In *Sulfolobus acidocaldarius*, three USP homologs are encoded in the genome and we designated them UspA (Saci_0887), UspB (Saci_1357) and UspC (Saci_1640), which for clarity we will call here *Sa*UspA, *Sa*UspB, and *Sa*UspC, respectively. We recently identified *Sa*UspA as an important interaction partner of PP2A, one of the two phosphatases of *S. acidocaldarius* (Ye et al., 2020), and here, we focused on the biochemical characterization of *Sa*UspA and investigated its physiological role in *S. acidocaldarius*. Our results indicate that *Sa*UspA belongs to the ATP-binding USP group and that ATP-binding by *Sa*UspA is important for its interaction with the threonine/serine phosphatase PP2A. Our results also showed that *Sa*UspA plays a role in the adaptation to high salinity stress.

## Materials and Methods

### Strains and Growth Conditions

*S. acidocaldarius* strains (MW001 and derived mutants) used in this study (Supplementary Table S1) were cultivated in Brock’ basal medium supplemented with 0.1% (w/v) NZ-amine, 0.2% (w/v) dextrin and 10 μg/mL uracil (Brock et al., 1972; Wagner et al., 2012). For *S. acidocaldarius* strains containing plasmids, the same medium without uracil was used. *E. coli* strains (Supplementary Table S1) were grown in Lysogeny broth (LB) medium or on LB agar plates with the appropriate antibiotics (50 μg/mL ampicillin, 25 μg/mL kanamycin or 30 μg/mL chloramphenicol). *E. coli* Top 10 was used for plasmids propagation. *E. coli* ER1281 was used to methylate plasmids for *S. acidocaldarius* transformation. *E. coli* Rosetta (DE3)/pLysS was used for overexpressing *S. acidocaldarius* proteins.

### Overexpression and Purification of *S. acidocaldarius* Proteins in *E. coli*

After the transformation of the expression plasmids (pSVA1037, pSVA1076, pSVA2009, pSVA5134, or pSVA5139) (Supplementary Table S1) into *E. coli* Rosetta (DE3)/pLysS, 20 mL of overnight culture of *E. coli* was inoculated in 2 L LB medium supplemented with the appropriate antibiotics and cultivated at 37°C and 140 rpm. At an OD_600_ of 0.5–0.6, the culture was cooled down and expression was induced with 500 μM Isopropyl β-D-1-thiogalactopyranoside (IPTG) at 16°C overnight. On the next day, cells were harvested by centrifugation and re-suspended in lysis buffer (50 mM Tris pH8, 150 mM NaCl) with DNaseI and protease inhibitor. Cells were lysed by using the French press, and the supernatant was collected after centrifugation at 4°C and 48,000 × *g* for 20 min. To remove *E. coli* proteins, the supernatant was heated in a water bath to 70°C for 20 min followed by ultracentrifugation at 236,000 × *g* for 45 min. The supernatant was used for His-tag or Strep-tag purification depending on the isolated protein. For *Sa*UspA and *Sa*UspA^G97A^, an extra ammonium sulfate precipitation step was performed to obtain nucleotide free protein. All proteins were applied to a Superdex 200 10/300 GL column or Superdex 200 16/600 column (GE Healthcare) equilibrated in lysis buffer before use.

### *In vitro* Protein Interaction Analysis

StrepII tagged *Sa*UspA or *Sa*UspA^G97A^ were used in interaction assays with His_6_ tagged PP2A. Shortly, 500 μL of each purified protein (0.5 mg/mL) were mixed and incubated at 55°C for 30 min. The mixture was applied to a 1000 μL PureSpeed (PS) tip with 20 μL IMAC resin (Mettler Toledo) as described in the manufacture’s instruction. 50 μL of lysis buffer containing 500 mM imidazole was used in the elution step. To prepare the ATP-bound sample, *Sa*UspA or *Sa*USP^G97A^ were incubated with 1 mM ATP in lysis buffer containing 5 mM MgCl_2_ at 25°C for 30 min before incubation with PP2A-his. Fractions from all assays were separated on SDS-PAGE and analyzed by Western blot using α-His and/or α-Strep antibodies.

### TNP-ATP Binding Assay

Binding of TNP-ATP [2′-(or-3′)-O-(Trinitrophenyl) Adenosine 5′-Triphosphate] was monitored by fluorescence increase upon binding to the protein using a Fluoromax-4 spectrofluorometer (Horiba). The excitation wavelength was set to 409 nm and the emission was detected at 540 nm. Slit widths for excitation and emission were set to 5 nm and 20 nm, respectively. Binding was measured by titration of 2.5 μM *Sa*UspA or *Sa*UspA^G97A^ with increasing TNP-ATP concentrations (0–30 μM) at 25°C in lysis buffer containing 5 mM MgCl_2_. Fluorescence was corrected for TNP-ATP fluorescence in the absence of proteins and fitted with the Hill equation: F = [TNP-ATP]^n^/(K${}_{\text{d}}^{\text{n}}$ + [TNP-ATP]^n^), with F = fluorescence, [TNP-ATP] = concentration of TNP-ATP, Kd = dissociation constant and *n* = hill constant.

### *In vitro* Phosphorylation

ArnC or ArnD were purified and *in vitro* phosphorylation assays with ArnC or ArnD were set up and performed as described by Reimann et al. (2012) in lysis buffer containing 1 mM MnCl_2_.

### Enzyme Assays of PP2A

Protein serine/threonine phosphatase activity of PP2A was determined by using the artificial p-peptides RRA(pT)VA as the substrate according to manufacturer’s instruction (Promega) (Reimann et al., 2013). The reaction was performed with 100 ng PP2A at 70°C in lysis buffer containing 1 mM MnCl_2_. The release of free phosphate was determined using the Malachite green assay (Van Veldhoven and Mannaerts, 1987).

### RNA Isolation and qRT-PCR

10 mL *S. acidocaldarius* culture was collected from the respective indicated growth conditions and time points, and total RNA was isolated with TRIzol (Invitrogen). Chromosomal DNA was removed from total RNA samples and cDNA was synthesized according to the standard protocol of the QuantiTect Reverse Transcription Kit (Qiagen). cDNA samples were analyzed with the qPCRBIO SyGreen Mix (PCR Biosystems) and Rotor-GeneQ Real-time PCR cycler (Qiagen). *secY* was used as the reference to normalize the qRT-PCR data. For analysis, at least three biological replicates and two technical replicates were used.

### Construction of Markerless In-Frame Deletion Mutant for *saUspA* in *S. acidocaldarius*

The markerless in-frame deletion mutant of *saUspA* (*saci_0887*) was constructed as described by Wagner et al. (2012). To construct the deletion mutant plasmid pSVA5113, a PCR was performed to obtain an overlap fragment of 549 bp of the upstream region and of 568 bp of the downstream region using primers 7350/7351 and 7352/7353 (see Supplementary Table S2). The overlap fragment was cloned into pSVA407. After sequencing, the plasmid was methylated in *E. coli* ER1821 and transformed into *S. acidocaldarius* MW001. Transformants were selected with first selection gelrite plates lacking uracil [0.6% gelrite (w/v)] and second selection gelrite plates containing uracil (10 μg/mL) and 5-FOA (100 μg/mL). Obtained colonies were screened for the deletion and positive clones were sequenced.

### Assays for Cell Survival and Aggregation Under UV Stress

UV treatment of *S. acidocaldarius* was performed as described by Wolferen et al. (2015). To determine cell survival rates, 10 mL culture (OD_600_ 0.2–0.3) was poured in plastic petri dishes and treated with different doses of UV light (75, 125, and 200 J/m^2^) (254 nm; Spectroline UV cross-linker). A culture without UV treatment was used as control. All the cultures were serially diluted in medium and spread on solid gelrite plates. Plates were cultivated at 75°C for 3–4 days until colonies were visible. Colonies from UV treated and non-UV treated samples were counted. To quantify the cell survival rates, data from at least three independent experiments was used. For analyzing ups pili dependent cell aggregation, a dose of 75 J/m^2^ UV light was used. Samples without UV treatment were used as the control. After UV treatment, samples were cultivated at 75°C for 3 h and analyzed with phase-contrast microscopy.

### Nutrient Starvation Assays and Western Blots

Cells were grown in nutrient rich medium (Brock’ basal medium with NZ-amine and dextrin) and at an OD_600_ of 0.4–0.5, cells were harvested by centrifugation at 75°C for 10 min and the supernatant was discarded. Cell pellets were resuspended in an equal volume of pre-warmed Brock’ basal medium without NZ-amine and dextrin and further cultivated at 75°C and 120 rpm. Samples were harvested at indicated time points.

Protein samples were separated by SDS-PAGE and transferred to a polyvinylidene difluoride (PVDF) membrane (Roche). The membrane was blocked in blocking buffer with 0.2% I-Block^TM^ (Thermo Fisher) for 1 h, followed by incubation with the primary antibody α-FlaB (Eurogentec) (Lassak et al., 2012) overnight. After incubation with the secondary anti-rabbit-HRP antibody (Invitrogen) for 1 h, chemiluminescent signals were visualized by ECL Chemocam Imager (INTAS) with Clarity Western ECL blotting substrate (Bio-Rad).

### Motility Assays

Motility assays were performed as described by Lassak et al. (2012). The respective *S. acidocaldarius* cultures were grown overnight till an OD_600_ of 0.4–0.5. 5 μL of each respective culture was spotted on semi-solid gelrite plates (0.15% gelrite (w/v)). Plates were incubated in closed plastic storage boxes at 75°C for 4–5 days before they were scanned.

## Results

### *Sa*UspA Interacts With PP2A

**FIGURE 1:**
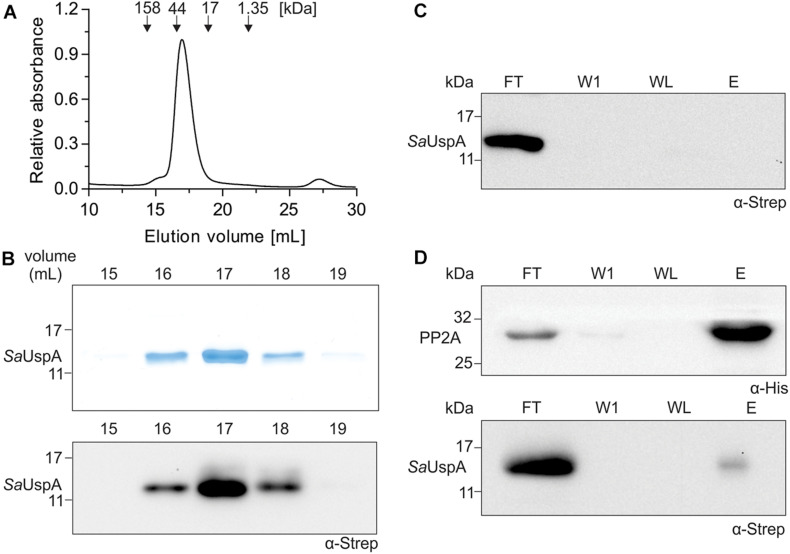
*Sa*UspA is purified as a dimer and interacts with PP2A. **(A)** Purified Strep II-tagged *Sa*UspA was analyzed on a Superdex 200 10/300 GL column. Molecular weight standard of 158, 44, 17, and 1.35 kDa are indicated. **(B)** Fractions from **(A)** were analyzed by SDS-PAGE (upper panel) and Western blot with α-Strep antibody (lower panel). **(C)** Strep II-tagged *Sa*UspA was applied to Ni-NTA resin, the resin was washed and eluted with buffer containing 10 and 500 μM imidazole, respectively. Fractions were analyzed by Western blot with α-Strep antibody. FT, flowthrough; W1, first wash fraction; WL, last wash fraction; E, elution fraction. **(D)** *In vitro* interaction assays for PP2A and *Sa*UspA. His-tagged PP2A and StrepII-tagged *Sa*UspA were mixed and a co-purification was performed as in **(C)**. All experiments were repeated at least three times and representative figures are shown.

**FIGURE 2:**
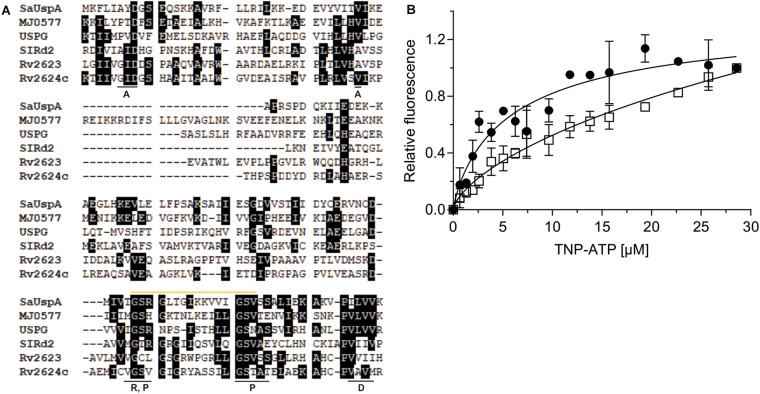
*Sa*UspA is an ATP-binding protein. **(A)** Multiple sequence alignment of *Sa*UspA and ATP-binding USP proteins including *E. coli* UspG, *M. jannaschii* MJ0577, *N. benthamiana* SlRd2, *M. tuberculosis* Rv2623 and Rv2624c. Conserved residues were marked with black boxes. Residues underlined with a black bar indicate residues important for dimerization (D) or residues interacting with the adenine (A), phosphate (P) or ribose (R) moieties of ATP are indicated. The Walker or P-loop motif [G-2x-G-9x-G-G(S/T)] responsible for ATP-binding is indicated by the yellow bar. **(B)** ATP-binding assays of *Sa*UspA. The relative increase in fluorescence upon ATP-binding was determined by titrating 2.5 μM *Sa*UspA^WT^ (WT) (black circle) or *Sa*UspA^G97A^ (G97A) (open square) with increasing concentrations of TNP-ATP. Data was obtained from at least three independent experiments and binding curves were fitted to the Hill equation.

*Sa*UspA was recently identified as an interaction partner of the serine/threonine phosphatase PP2A during early nutrient starvation in *S. acidocaldarius* (Ye et al., 2020). Here, we set-out to further characterize *Sa*UspA and its interaction with PP2A. To that end, C-terminally his_6_-tagged PP2A (PP2A-his) and C-terminally StrepII-tagged *Sa*UspA (*Sa*UspA-Strep) were purified. Size exclusion chromatography of *Sa*UspA (molecular weight: 16.2 kDa) resulted in a monodisperse peak eluting at a volume corresponding to a molecular weight of ∼ 30 kDa (Figures 1A,B) suggesting that *Sa*UspA forms a dimer. Dimerization of USP proteins occurs via a small C-terminal dimerization domain with a conserved sequence (See Figure 2A; Gutiérrez-beltrán et al., 2017). Overexpression in *E. coli* of a *Sa*UspA variant which lacked the 5 C-terminal residues forming the dimerization domain (*Sa*UspA^Δ*dim*^) was not successful, which implied that the conserved dimerization domain is also critical for the stability of *Sa*UspA. To further confirm the previously observed interaction between PP2A and *Sa*UspA, a co-purification assay with PP2A-his and *Sa*UspA-Strep was performed on Ni-NTA resin. *Sa*UspA-Strep did not bind to the Ni-NTA resin by itself (Figure 1C), but *Sa*UspA was identified in the elution fraction in the presence of PP2A-his (Figure 1D), further demonstrating that both proteins interact.

### *Sa*UspA Is an ATP-Binding Protein

A multiple protein sequence alignment with five USP proteins that have previously been shown to bind ATP, *M. jannaschii* MJ0557, *E. coli* UspG, *Nicotiana benthamiana* SIRd2, *M. tuberculosis* Rv2623 and Rv2624c was made (Zarembinski et al., 1998; Drumm et al., 2009; Jia et al., 2016; Gutiérrez-beltrán et al., 2017). This showed that the conserved ATP-binding Walker or P-loop motif [G-2X-G-9X-G(S/T)] and the C-terminus dimerization domain are conserved between *Sa*UspA and these other five USP proteins (Figure 2A), suggesting that *Sa*UspA belongs to the ATP-binding members of the USP family. To investigate whether *Sa*UspA binds ATP, ATP-binding assays were performed using the fluorescent ATP analog TNP-ATP. To determine the binding affinity, 2.5 μM of *Sa*UspA was titrated with 1–30 μM TNP-ATP. Normalized fluorescence intensities were plotted against the TNP-ATP concentration and data was fitted with the Hill equation (Hill coefficient of 0.84) resulting in a dissociation constant of 7 +/− 2 μM (Figure 2B), demonstrating that *Sa*UspA could bind TNP-ATP. *Sa*UspA was also tested for ATP hydrolysis, but ATP hydrolysis was below our detection limits. In *M. tuberculosis*, The D15E and G117A mutants in Rv2623 showed 34% and 21% ATP binding compared to wild type Rv2623 (Drumm et al., 2009), and mutation of D17 into E and G119 to alanine abolished Rv2624c’s ATP binding ability (Jia et al., 2016). To test whether these two residues are also important for ATP binding in *Sa*UspA, the corresponding amino acids in *Sa*UspA (D8 and G97) were mutated to alanine. No protein could be obtained when *Sa*UspA^D8A^ was expressed in *E. coli*, suggesting this mutation either destabilized the protein or that *Sa*UspA is stabilized by ATP binding and the mutated protein can no longer bind ATP. However, *Sa*UspA^G97A^ was successfully expressed and purified. *Sa*UspA^G97A^ bound TNP-ATP-binding with a 50-fold lower dissociation constant of around 350 μM (Figure 2B), demonstrating that G97 of *Sa*UspA plays a role in ATP binding.

### *Sa*UspA Is Phosphorylated by ArnC

**FIGURE 3:**
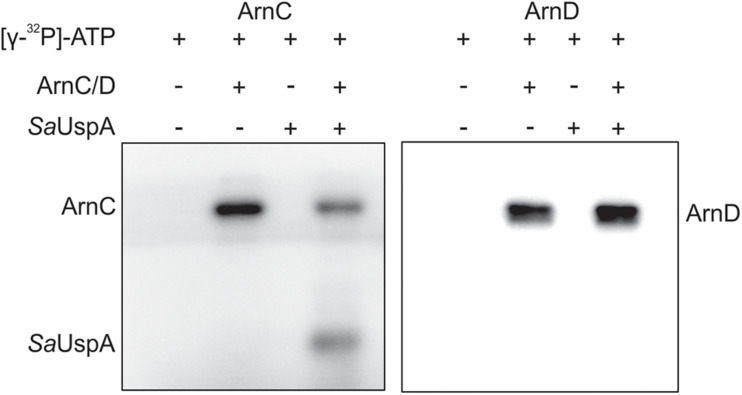
*In vitro* phosphorylation assays of *Sa*UspA. Purified *Sa*UspA was incubated at 55°C using Mn^2+^ as cofactor in the presence or absence of the kinases ArnC or ArnD and [γ−^32^P] ATP.

In bacteria and plants, several members of the USP superfamily are phosphorylated, however, only a few kinases responsible for phosphorylation were identified (Freestone et al., 1997; Lenman et al., 2008; Merkouropoulos et al., 2008; Glass et al., 2017; Gutiérrez-beltrán et al., 2017; Potel et al., 2018). In *S. acidocaldarius*, a number of protein kinases have been characterized and ArnC and ArnD are relatively well studied (Reimann et al., 2012; Hoffmann et al., 2017, 2019; Bischof et al., 2019; Maklad et al., 2020). To investigate whether *Sa*UspA can autophosphorylate or is phosphorylated by ArnC or ArnD *in vitro*, *Sa*UspA was incubated with or without ArnC or ArnD and [γ−^32^P] ATP (Figure 3). When *Sa*UspA was incubated with [γ−^32^P] ATP, no autophosphorylation could be detected, whereas, as described previously (Reimann et al., 2012) autophosphorylation was detected for both ArnC and ArnD. Incubation of *Sa*UspA with ArnC resulted in phosphorylation of *Sa*UspA and loss of phosphorylation of ArnC, while no effects could be observed when *Sa*UspA was incubated with ArnD. Thus, *Sa*UspA did not show autophosphorylation activity *in vitro*, but could be phosphorylated by ArnC.

### Binding of ATP by *Sa*UspA Enhances Its Interaction With PP2A

**FIGURE 4:**
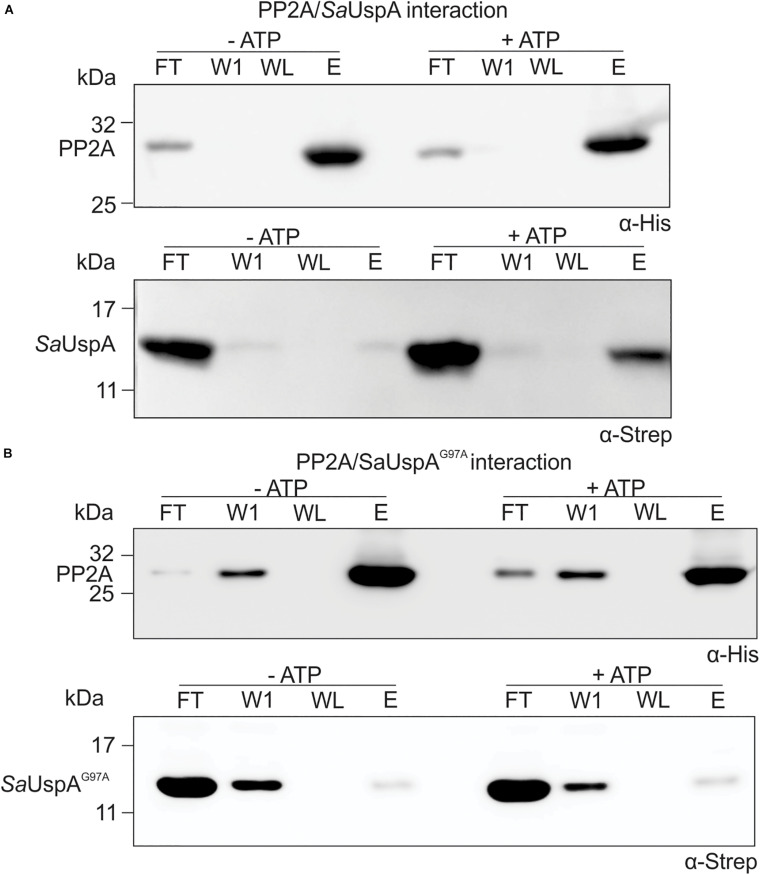
ATP-binding to *Sa*UspA increases the interaction with PP2A. *In vitro* interaction assays for PP2A and *Sa*UspA. **(A)** PP2A-his and *Sa*UspA-Strep were mixed and applied to Ni-NTA resin, the resin was washed and eluted with buffer containing 10 and 500 μM imidazole, respectively. Fractions were analyzed by Western blot with α-His and α-Strep antibody. **(B)** As in **(A)** but using *Sa*UspA^G97A^. FT, flowthrough; W1, first wash fraction; WL, last wash fraction; E, elution fraction. All experiments were repeated at least three times and representative figures are shown.

Since *Sa*UspA binds ATP and interacts with PP2A in the *in vitro* assays, it was tested whether the interaction between PP2A and *Sa*UspA was modified by ATP binding. Upon the addition of ATP, a clear increase of *Sa*UspA was detected in the elution fraction of the interaction assay when compared to the sample without ATP (Figure 4A). This indicated that ATP-binding of *Sa*UspA enhanced its interaction with PP2A. When the assays were performed with the ATP-binding deficient mutant *Sa*UspA^G97A^ (Figure 4B), interaction between *Sa*UspA^G97A^ and PP2A was still observed, but no increase could be observed upon the addition of ATP, further demonstrating that ATP-binding to *Sa*UspA stimulates binding to PP2A.

### *Sa*UspA Stimulates the Phosphatase Activity of PP2A

**FIGURE 5:**
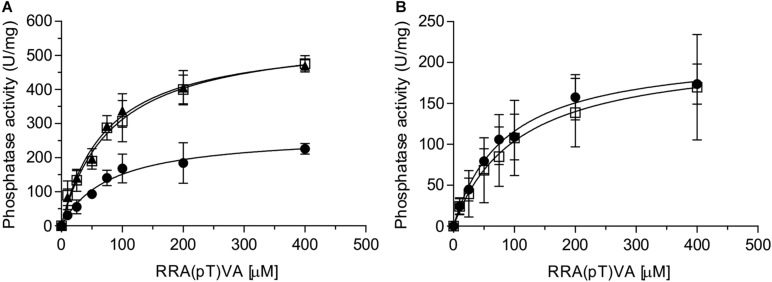
*Sa*UspA stimulates the phosphatase activity of PP2A. The phosphatase activity of PP2A was determined in the absence (black circle) or presence of *Sa*UspA (3 μM) with (black triangle) or without (open square) ATP **(A)**, or AbfR1 (3 μM) (open square) **(B)**. All experiments were performed at 70°C in biological triplicates.

Eukaryotic PP2A is a heterotrimeric complex containing a scaffolding, a regulatory and a catalytic subunit (Shi, 2009). The regulatory subunit is involved in regulating the activity of PP2A (Drewes et al., 1993; Gong et al., 1994; Santoro et al., 1998). In addition, a large number of proteins have been identified which interact with one or more subunits or even a specific PP2A, thereby regulating PP2A’s function (Dozier et al., 2004; McConnell et al., 2007). PP2A is the homolog of the eukaryotic PP2A catalytic subunit, but no homologs of the scaffolding or regulatory subunits have been identified in archaea. Thus, it seems likely that proteins like *Sa*UspA, which were previously identified in the pull-down assay (Ye et al., 2020) regulate the activity of PP2A. To investigate whether *Sa*UspA regulates the activity of PP2A, the PP2A phosphatase activity was determined at different concentrations of an artificial phospho-peptide (Figure 5A). As observed previously (Reimann et al., 2013), the phosphatase activity of PP2A had a Km of 84 +/− 16 μM and a Vmax of 274 +/− 16 U/mg, respectively. Remarkably, in the presence of *Sa*UspA, no effect was observed on the Km, but the Vmax increased to 583 +/− 55 U/mg. To our knowledge, this is the first observation of the regulation of the activity of an archaeal phosphatase by another protein. Since ATP enhances the interaction between *Sa*UspA and PP2A, ATP might affect the stimulation of PP2A by *Sa*UspA. However, no effects were observed in the presence of ATP. Most likely PP2A and *Sa*UspA already form a complex at the concentration used in this assay, and ATP-dependent stimulation of the interaction occurs only at lower concentrations. To ensure that the observed effect is specific for the interaction between *Sa*UspA and PP2A, a similar experiment was performed with PP2A and AbfR1 (Figure 5B). AbfR1 is a regulator of biofilm and motility but was not identified as a strong interaction partner in the previous pull-down assays (Li et al., 2017; Ye et al., 2020). When PP2A was assayed in the presence of AbfR1, its phosphatase activity did not show significant differences, which showed that the stimulating effect on PP2A activity was specific to *Sa*UspA.

### Transcription of *saUspA* Is Growth Phase Independent and Neither Induced by Nutrient Starvation nor by High Salinity Stress

**FIGURE 6:**
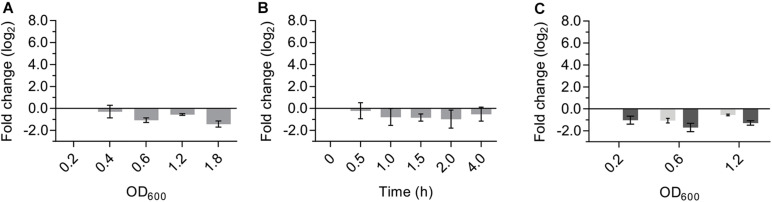
Transcription levels of *saUspA* do not change in response to different stress conditions. **(A)** *S. acidocaldarius* was grown in nutrient-rich medium and cell samples were taken at different OD_600_. OD_600_ of 0.2, 0.4, 0.6, and 1.2 belong to the exponential phase of cell growth. OD_600_ of 1.8 belongs to the stationary phase of cell growth. Samples of OD_600_ 0.2 were used as the control for analysis. **(B)** *S. acidocaldarius* was grown in nutrient-depleted medium and cell samples were taken at different time points. Samples taken at t_0_ were used as the control for analysis. **(C)** *S. acidocaldarius* was grown in nutrient-rich medium in the presence (dark gray) and absence (light gray) of NaCl (400 mM), and cell samples were taken at OD_600_ 0.2, 0.6, and 1.2. Samples of OD_600_ 0.2 in the absence of NaCl were used as the control for analysis. Transcription level of *saUspA* was analyzed by qRT-PCR. Relative transcription level was normalized to *secY*. The values represent fold changes (mean ± SD) compared with the control from biological triplicates.

Universal stress proteins in bacteria and eukarya are important for many different responses to stress, but the role of USP proteins in archaea is currently still unknown. To determine the physiological role of *Sa*UspA, the transcriptional activity of the *saUspA* gene under different conditions was studied. Cells of *S. acidocaldarius* MW001 growing in nutrient-rich medium were collected at different OD_600_ and RNA was isolated and analyzed by qPCR. The transcript levels of *saUspA* did not change significantly during the different stages of the growth curve and were, in contrast to other studied *usp* genes (Nystrdm and Neidhardt, 1992; Liu et al., 2007), not induced in the stationary phase (Figure 6A). Also under nutrient depletion (Figure 6B) or high salinity stress (400 mM NaCl) (Figure 6C), no significant up- or down regulation of *saUspA* was observed. Also previous transcriptome data of *S. acidocaldarius* (Götz et al., 2007) showed that exposure to 200 J/m^2^ UV irradiation did not change the transcription of *saUspA* significantly.

### Effect of *saUspA* Deletion on *S. acidocaldarius* Cells Responding to Different Stress Conditions

**FIGURE 7:**
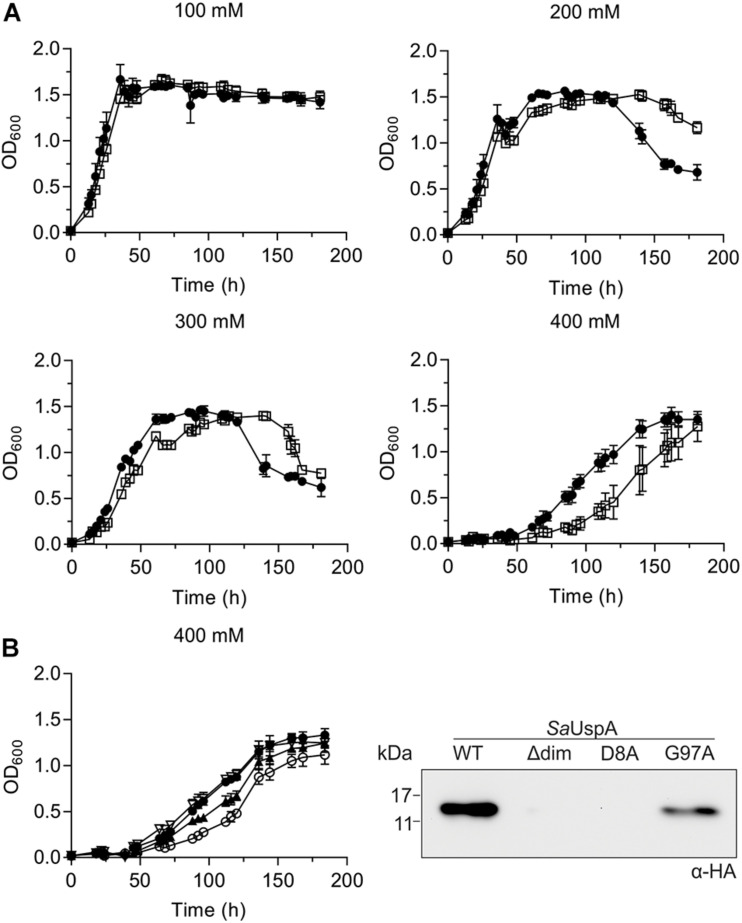
The *saUspA* deletion mutant is affected during growth on high salt concentrations. **(A)** Growth curves of *S. acidocaldarius* MW001 (black circle) and the Δ *saUspA* mutant (open square) in the presence of 100, 200, 300, and 400 mM NaCl. The growth curves were performed in triplicate and standard deviations are shown. **(B)** Left panel: complementation of the Δ*saUspA* mutant growth defect in presence of high salinity with plasmid-based expressed *Sa*UspA and its ATP-binding deficient mutant from its native promoter. Empty vector (open circle) and plasmids containing *saUspA*^WT^ (open triangle) and *saUspA*^G97A^ (black triangle) were used to complement the Δ*saUspA* mutant. MW001 containing empty vector (black circle) was used a control. Cells were grown in the presence of 400 mM NaCl. Right panel: plasmid-based expression of HA-tagged *Sa*UspA^WT^ (WT), *Sa*UspA^Δ*dim*^ (Δdim), *Sa*UspA^D8A^ (D8A) and *Sa*UspA^G97A^ (G97A) in the *S. acidocaldarius* Δ*saUspA* mutant was confirmed by western blot analysis. *S. acidocaldarius* cells were grown in nutrient-rich medium without uracil and cell samples were taken at OD_600_ of 0.4, which was analyzed by Western blot with α-HA antibody.

To identify a physiological role of *Sa*UspA, a markerless in-frame deletion mutant was constructed. Deletion of *saUspA* did neither show an effect on normal growth nor any effect on cell survival and growth during UV stress or during growth on heavy metals (Supplementary Figures S1–S3). It has been shown previously that *S. acidocaldarius* can grow in NaCl concentrations up to 400 mM (Meyer et al., 2011). MW001 and the Δ*saUspA* strain were both cultivated in 100 to 400 mM NaCl (Figure 7A). Increasing concentrations of NaCl slowed the growth of both strains and at 400 mM NaCl, the Δ*saUspA* mutant grew significantly slower than the wild type strain MW001. Furthermore, the Δ*saUspA* mutant showed a clear increase in the lag phase. Microscopic analysis of the strain under these conditions did not reveal any changes in size or morphology of the cells compared to the MW001 strain. Thus the Δ*saUspA* stain shows decreased growth under high salinity condition. To test whether this phenotype could be complemented by expression of *Sa*UspA, a plasmid which expressed C-terminally HA-tagged *Sa*UspA, *Sa*UspA^Δ*dim*^, *Sa*UspA^D8A^, or *Sa*UspA^G97A^ from the *saUspA* promotor was constructed and transformed to the *S. acidocaldarius* Δ*saUspA* strain. No expression of the *Sa*UspA^Δ*dim*^ and *Sa*UspA^D8A^ proteins could be detected using Western blots analysis with an anti-HA antibody (Figure 7B), which confirmed that ATP binding and dimerization are important for the stability of this protein. *Sa*UspA^G97A^ which showed reduced ATP binding was also expressed at a lower level than WT *Sa*UspA (Figure 7B). Expression of *Sa*UspA fully complemented the growth defect of the Δ*saUspA* mutant whereas the reduced ATP binding mutant *Sa*UspA^G97A^ could only restore the growth defect partially (Figure 7B).

### Deletion of *saUspA* Affects the Timing of FlaB Expression

**FIGURE 8:**
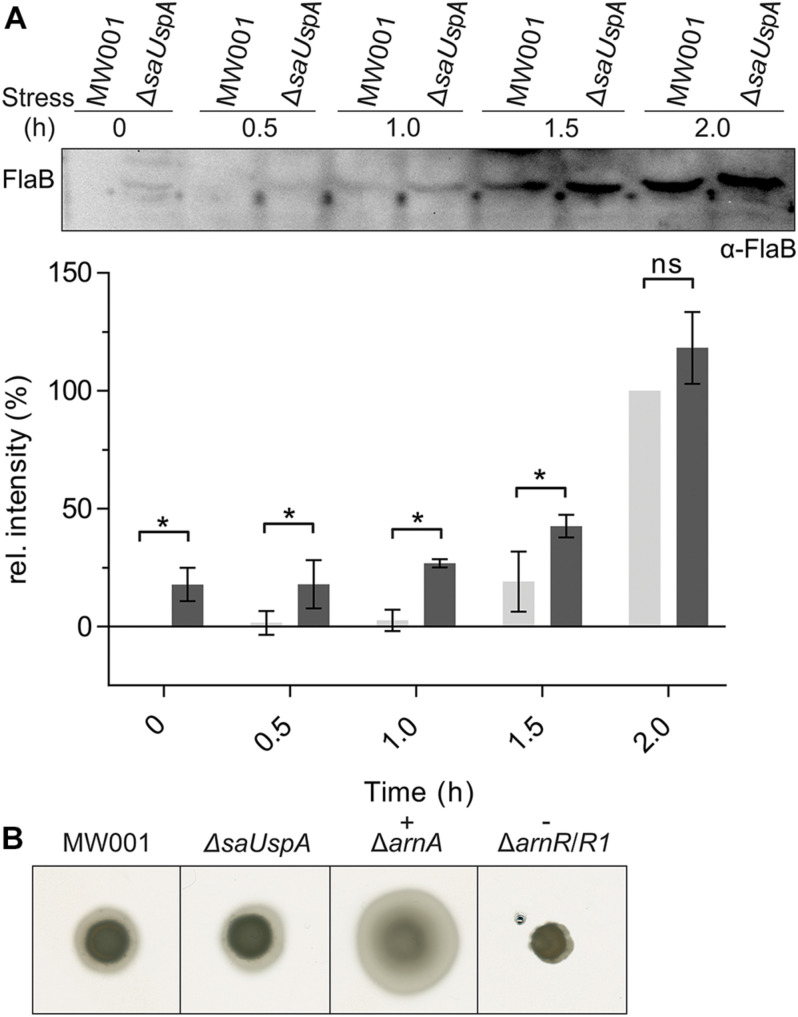
Effect of *saUspA* deletion on FlaB expression and motility. **(A)** Time course of FlaB expression during 2 h growth in nutrient-depleted medium. Wild type and Δ*saUspA* mutant cells were grown in nutrient-depleted medium for 2 h and cell samples were taken at different time points. The FlaB expression level was analyzed by Western blot with an α-FlaB antibody. A representative Western blot is shown. A quantification of Western blots of three biological replicates is shown for MW001 (light gray) and the Δ*saUSPA* mutant (dark gray) below the blot. The expression levels of FlaB at 0 and 2 h for strain MW001 were used as minimal and maximal values for data normalization. Significant differences between MW001 and Δ*saUSPA* mutant (*p*-value < 0.05) are indicated by an asterisk while ns indicates no significance (*p*-value > 0.05). **(B)** Motility assay. Same amounts of cells were spotted on semi-solid gelrite plates. After incubating at 75°C for 5 days, the plates were scanned and recorded. Δ*arnA* and Δ*arnR/R1* were used as hyper-motile and non-motile control, respectively. The figures shown here were representative examples of biological triplicates.

One of the phenotypes of the *Δpp2a* strain is hyper-motility (Reimann et al., 2013). Motility in archaea is driven by the archaellum (Jarrell and Albers, 2012), which in *S. acidocaldarius* is expressed under starvation conditions (Lassak et al., 2012). To test whether *Sa*UspA plays a role in the archaellum regulation network in *S. acidocaldarius*, expression of the archaellin FlaB was analyzed by Western blot analysis during the first 2 h after induction of starvation conditions (Figure 8A and Supplementary Figure S4). FlaB expression increased during nutrient starvation in both the wild type strain and the Δ*saUspA* mutant strain. Compared with the WT strain, increased FlaB expression was observed in Δ*saUspA* mutant at 0, 0.5, 1.0, and 1.5 h. However, at time point 2.0 h, no significant difference in FlaB expression was observed. Thus, deletion of *saUspA* leads to early expression of FlaB during nutrient starvation, however, the final expression level of FlaB was not affected. In addition, motility of Δ*saUspA* mutant on semi-solid gelrite plates was analyzed (Figure 8B), and as expected from the similar FlaB levels after 2 h, no differences in motility were observed. Thus, deletion of *saUspA* did not have significant effects on the motility of *S. acidocaldarius* cells.

## Discussion

*Sa*UspA, a member of the universal stress protein family, was recently identified as a binding partner of the serine/threonine phosphatase PP2A (Ye et al., 2020), during the early nutrient starvation response of *S. acidocaldarius*. PP2A is the homolog of the eukaryotic PP2A catalytic subunit, a heterotrimeric complex containing also a scaffolding, a regulatory and a catalytic subunit (Drewes et al., 1993; Gong et al., 1994; Santoro et al., 1998; Shi, 2009). This heterotrimeric complex interacts with different proteins which interact with one or more subunits or even a specific PP2A, thereby regulating PP2A’s function (Dozier et al., 2004; McConnell et al., 2007). In previous studies, it was proposed that PP2A is involved in the archaellum regulatory network during normal growth and starvation when expression of the archaellum is strongly induced (Reimann et al., 2013). In bacteria, proteins of the USP superfamily were previously shown to be involved in the adaptation to various nutrient starvation conditions and also functioned as regulators in motility (Nystrom and Neidhardt, 1994; Gustavsson et al., 2002; Nachin et al., 2005). Based on these findings, we set out to study whether *Sa*UspA might be involved in nutrient starvation signaling or motility in *S. acidocaldarius* by modulating the activity of PP2A.

### *Sa*UspA Is Stabilized by ATP-Binding and Dimerization

As was previously observed for other USPs, *Sa*UspA formed a stable homodimer. Deletion of the C-terminal dimerization domain resulted in strongly reduced levels of *Sa*UspA, both when expressed in *E. coli* or in *S. acidocaldarius*, Indeed, also truncation of six amino acids containing the C-terminal dimerization motif from *E coli* UspG, abolished dimer formation and resulted in a protein more sensitive to proteolysis (Weber and Jung, 2006). In *S*. *lycopersicum*, SlRd2 was still expressed after truncation of the dimerization motif but, its function in LiCl resistance and interaction with SlCipk6 was lost (Gutiérrez-beltrán et al., 2017). A similar observation was made for ATP binding. Previous studies showed that the substitution of the two conserved residues D17 and G109 in the ATP binding of Rv2624c resulted in reduced ATP-binding affinity and reduced thermal stability (Jia et al., 2016). Whereas WT *Sa*UspA could be stably overexpresssed, overexpression of *Sa*UspA with a mutation in D8 could neither be detected in *S. acidocaldarius* nor when the protein was heterologously expressed in *E. coli*. Mutagenesis of G97 resulted in reduced expression of *Sa*UspA in both *S. acidocaldarius* and in *E. coli* and strongly reduced ATP binding. This shows that for *Sa*UspA both ATP binding and dimerization are important for the stability of the protein. Although protein phosphorylation was found in some members of the USP superfamily (Freestone et al., 1997; Weber and Jung, 2006; Gutiérrez-beltrán et al., 2017; Potel et al., 2018), the physiological role is poorly understood. In *M. tuberculosis*, Rv2623 was phosphorylated at threonine 237, which was recognized by one FHA domain of Rv1747. The substitution of threonine 237 with a non-phosphorylatable alanine decreased their interaction and abolished their function in regulation growth (Glass et al., 2017). However, although high affinity binding of ATP to *Sa*UspA was observed, neither ATP hydrolysis nor autophosphorylation was observed. In addition, no phosphorylated form of *Sa*UspA could be identified in previous phosphoproteomic studies of *S. acidocaldarius* (Reimann et al., 2013). *In vitro* phosphorylation of *Sa*UspA by ArnC but not ArnD was observed, but the relevance of this observation is currently still unknown.

### The Interaction Between PP2A and *Sa*UspA Is Enhanced by ATP Binding and Stimulates the Phosphatase Activity of PP2A

Within this study, the previously observed interaction between PP2A and *Sa*UspA (Ye et al., 2020) was confirmed *in vitro*. Remarkably, this interaction occurred with higher affinity in the presence of ATP. Since *Sa*UspA has no autophosphorylation activity, the increased interaction is caused by binding of ATP to *Sa*UspA and not by autophosphorylation of *Sa*UspA. We have previously proposed that the proteins that interact with PP2A, a conserved putative ATP/GTP binding protein (Saci_1281), the archaellum regulators ArnA and ArnB and *Sa*UspA might regulate the activity of PP2A and thus might regulate different cellular processes (Ye et al., 2020). Here it is demonstrated that binding of *Sa*UspA to PP2A stimulates the phosphatase activity of PP2A. Although members of the USP superfamily have been studied for years, the mechanism by which they function are still largely unknown. We here propose that one possible mechanism by which UPSs influence the stress response is by interacting and modulating the activity of phosphatases.

### *Sa*UspA Plays a Role in Nutrient Starvation and High Salinity Stress

To determine in which cellular processes *Sa*UspA plays a role, the effect of *Sa*UspA on different processes was studied. Remarkably, unlike the stress-dependent transcription stimulation of *usp* genes in bacteria (Vollmer and Bark, 2018), the transcription level of *saUspA* was constant in *S. acidocaldarius* during growth in nutrient-rich medium and was not induced by nutrient starvation, high salinity and UV stress.

Deletion of *saUspA* did also not affect cell survival, growth during UV stress or during growth on heavy metals. A clear effect was observed under nutrient starvation conditions, where Δ*saUspA* cells expressed FlaB much earlier even under non-nutrient starvation condition, which indicated that the deletion of *saUspA* led to the induction of signaling pathways that normally occur during nutrient limitation. Remarkably, this was also observed in the Δ*pp2a* deletion strain (Reimann et al., 2013), suggesting that the UspA-PP2A interaction might be required for the response to nutrient starvation. Furthermore, the Δ*saUspA* strain grew significantly slower on high salinity medium. The growth defect could be complemented by the plasmid-based expression of *Sa*UspA, demonstrating that *Sa*UspA also plays a role during salinity stress. Remarkably, the expression levels of *Sa*UspA are constant under these high salt stress conditions, demonstrating that the presence, but not the upregulation of *Sa*UspA is important for the function of *Sa*UspA under these stress conditions. Furthermore, effects of the deletion of *saUspA* might be compensated by the two other USP superfamily members, *Sa*UspB and *Sa*UspC. These proteins were, however, not identified as interaction partners of PP2A. There are many possible mechanisms by which *Sa*UspA might be involved in resistance to high concentrations (∼400 mM) of NaCl, but the mechanism in *S. acidocaldarius* is unknown. For example, in *E. coli* the UspA family protein UspC binds to and stabilizes the two component system KdpD/KdpE which controls the induction of the high-affinity K^+^-transport system KdpFABC under conditions of salt stress (Heermann et al., 2009). Alternatively, *Sa*UspA might influence proteins involved in growth at high salinity via its interaction with PP2A. In, for example, the halophilic archaeon *M. portucalensis* FDF1T most proteins participating in uptake and synthesis pathways of the compatible solute betaine were phosphorylated including the glycine betaine BtaABC transporter and enzymes involved in the methionine transmethylation cycle and betaine biosynthesis (Wu et al., 2016).

## Data Availability Statement

The original contributions presented in the study are included in the article/Supplementary Material, further inquiries can be directed to the corresponding author.

## Author Contributions

XY, CD, and S-VA designed the experiments, analyzed the data, and wrote the manuscript. XY contributed to most of the experiments and figures. CD contributed to *in vitro* phosphorylation assays using radioactive-labeled ATP. All authors contributed to the article and approved the submitted version.

## Conflict of Interest

The authors declare that the research was conducted in the absence of any commercial or financial relationships that could be construed as a potential conflict of interest.
