# Supplementary material for: SaUspA, the Universal Stress Protein of Sulfolobus acidocaldarius Stimulates the Activity of the PP2A Phosphatase and Is Involved in Growth at High Salinity
Source: Front Microbiol. 2020 Nov 13;11:598821. doi: 10.3389/fmicb.2020.598821 (PMC7693658; doi:10.3389/fmicb.2020.598821)
Supplement: Supplementary file 1 [file Data_Sheet_1.PDF]

Supplementary materials of

"*Sa*UspA, the universal stress protein of *Sulfolobus acidocaldarius* stimulates the activity of the PP2A phosphatase and is involved in growth at high salinity"

Xing Ye, Chris van der Does and Sonja-Verena Albers

Deletion of *saUspA* doesn't affect cell growth under heavy metal stress and cell survival under UV stress

UV stress. To test the effect of the *saUspA* deletion on cell survival under UV stress, different doses of UV irritation (75, 125 and 200 J/m^2) were used and the cell survival rate was calculated (Fig. S1). Compared with the MW001 strain, there were no significant changes of the survival rate observed in the Δ*saUspA* mutant.

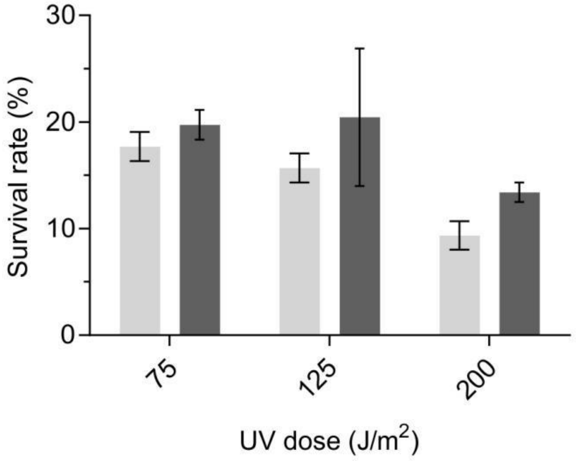

Fig. S1 Survival rate assays upon UV induction of Δ*saUspA* mutant.

S. acidocaldarius MW001 (light grey) and Δ*saUspA* mutant (dark grey) cells were treated with the indicated doses of UV. The percent of survival (mean ± SD) upon UV treatment was calculated from at least three independent experiments.

In *S. acidocaldarius*, cells can aggregate via UV induced pili to facilitate DNA exchange for survival under UV exposure, which can be visualized by microscopy (Wolferen et al., 2015). For MW001 and Δ*saUspA* mutant, similar amounts of cell aggregates were observed (Fig. S2A). Transcription levels of the *upsA* gene in *ups* gene cluster is always in line with the cell aggregation in *S. acidocaldarius* (Le et al., 2017). qPCR analysis showed similar transcripts levels of *upsA* were found in MW001 and the Δ*saUspA* mutant (Fig. S2B), which confirmed that deletion of *saUspA* did not affect cell aggregations in *S. acidocaldarius*.

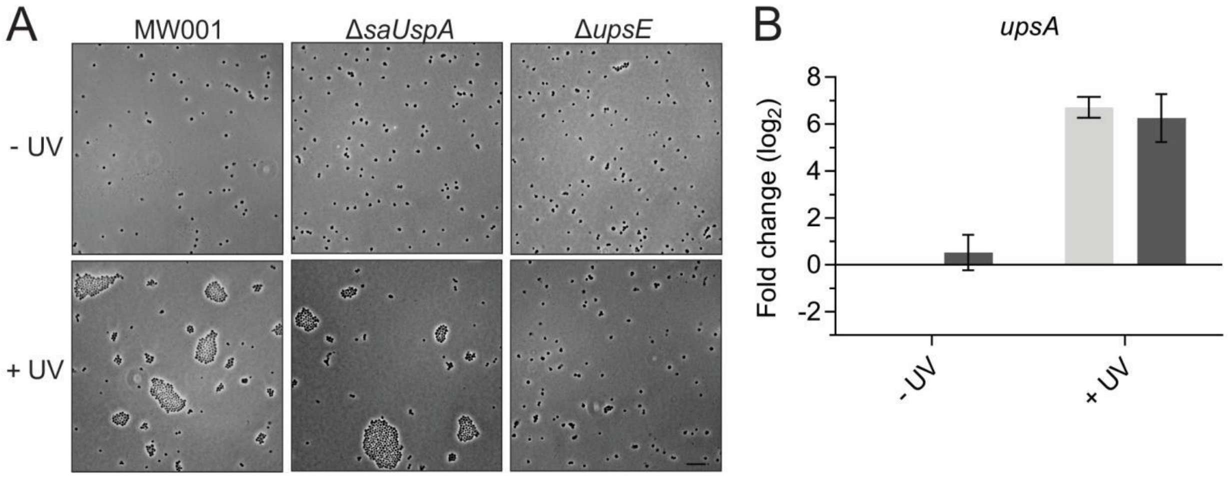

Fig. S2 (A) Aggregation assays with the Δ*saUspA* mutant. Non-UV treated cells (-UV) and cells 3 h after induction with UV light (75 J/m^2) (+UV) were visualized using phase-contrast microscopy. MW001 and Δ*upsE* were used as a positive and negative control, respectively (scale bar, 10 μm). **(B)** Transcription level of *upsA* 3h after UV treatment in MW001 (light grey) and Δ*saUspA* mutant (dark grey). Samples of non-UV treated MW001 were used as the control for analysis. Transcription level of *upsA* was analyzed by qRT-PCR. Relative transcription levels were normalized to *secY*. The values represent fold changes (mean ± SD) compared with the control from biological triplicates.

Heavy metal stress. To investigate the effect of heavy metal stress on growth of the Δ*saUspA* mutant, different concentrations of $NiSO_4$ were used (Fig.S3). There was no significant effect on the growth MW001 and the Δ*saUspA* mutant in nutrient-rich medium without $NiSO_4$. Growth of both strains grew slowly with the increase of $NiSO_4$, while there were no significant differences observed between them. So, deletion of *saUspA* did not affect the growth of *S. acidocaldarius* under heavy metal stress.

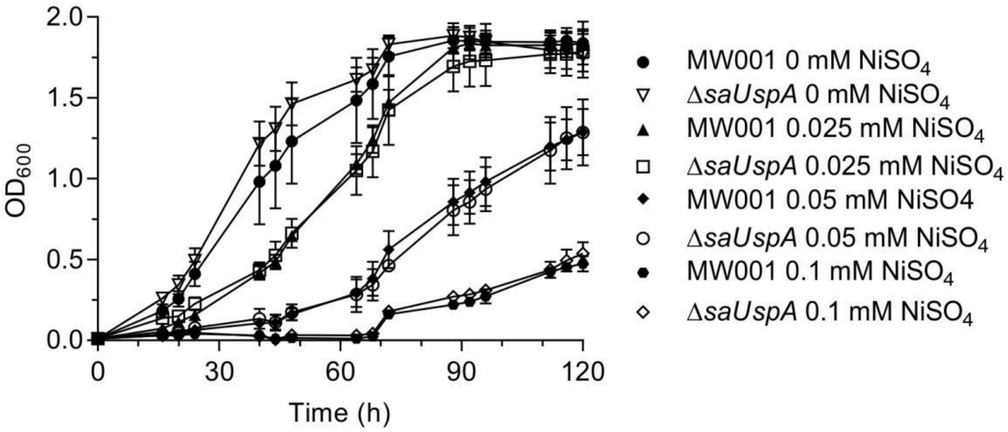

Fig. S3 Growth curves of *S. acidocaldarius* MW001 and Δ*saUspA* mutant in the presence different concentration of $NiSO_4$. The growth curves were performed in triplicate and standard deviations are shown.

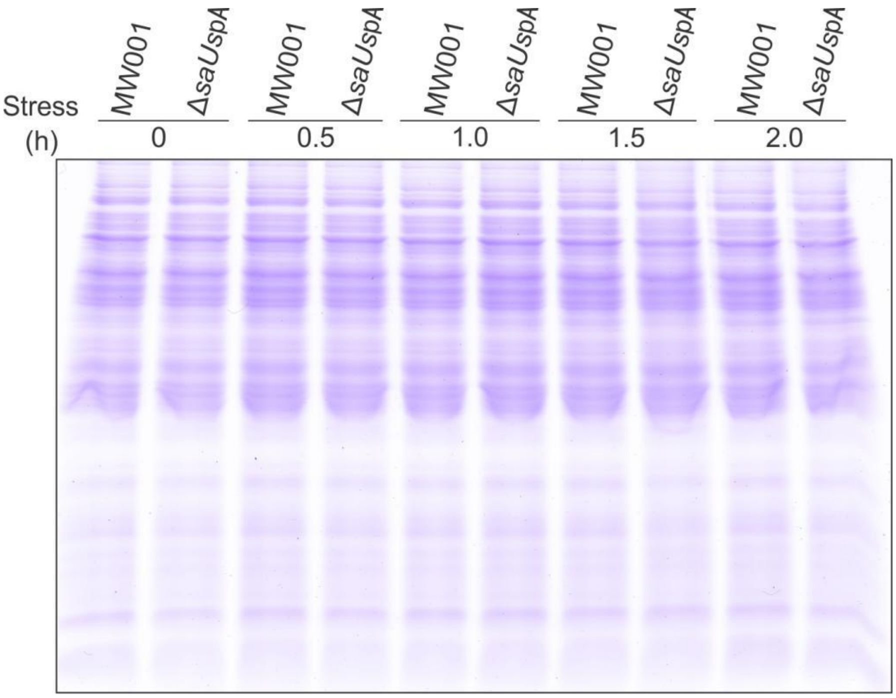

Fig. S4 Protein loading control for Fig 8.A. *S. acidocaldarius* MW001 and the Δ*saUspA* mutant were grown in nutrient starvation medium for 2 h. Samples were collected at different time points (0 h, 0.5 h, 1.0 h, 1.5 h and 2.0 h) and loaded on an SDS-PAGE.

Table S1 Strains and plasmids used in this study

Strains/Plasmids	Genotype	Source/Reference
**Strains**		
Escherichia coli		
Top10	F- mcrA Δ(mrr-hsdRMS-mcrBC) φ80lacZΔM15 ΔlacX74 nupG recA1 araD139 Δ(ara-leu)7697 galE15 galK16 rpsL(Str^R) endA1 λ^-	Invitrogen
Rosetta (DE3) /pLysS	F^- *ompT hsdS*$_B$(r_B^- m_B^-) *gal dcm* (DE3) pLysSRARE (Cam^R)	Novagen
ER1821	F- *glnV44 e14-(McrA-) rfbD1? relA1? endA1 spoT1? thi-1 Δ(mcrC-mrr)114::IS10*	New England Biolabs
Sulfolobus acidocaldarius		
MW001	*Sulfolobus acidocaldarius* DSM639 Δ*pyrE*	(Wagner et al., 2012)
MW812	MW001Δ*saci0887* (Δ*saUspA*)	This study
MW332	MW001Δ*saci1171*Δ*saci1180*(Δ*arnR*Δ*arnR1*)	(Lassak et al., 2012)
MW351	MW001 Δ*saci1210* (Δ*arnA*)	(Reimann et al., 2012)
MW109	MW001 Δ*saci*1494 (Δ*upsE*)	(Wolferen et al., 2015)
**Plasmids**		
pSVA1009	Saci1193 (*arnC*) with N-terminal His-tag cloned into pETDuet-1 with *BclI*/*BamHI*, *PstI* into MCSI	(Reimann et al., 2012)
pSVA1037	Saci0884 (*pp2a*)with C-terminal His-tag cloned into pETDuet-1 with *NcoI*, *BamHI* in MCSI	(Reimann et al., 2012)
pSVA1076	*Saci1694* (*arnD*) with C-termial His-tag cloned into pETDuet-1 with *Nco*I, *Pst*I in MCSI	(Reimann et al., 2012)
pVA2017	Saci0446 (*abfR1*) cloned into pETDuet-1 using *NotI* and *EcoRI* sites	(Li et al., 2017)
pSVA407	Gene targeting plasmid, pGEM-T Easy backbone, *pyrEFSSO* and *lacSSSO* cassette; single crossover method	(Wagner et al., 2012)
pSVA5113	In-frame deletion of *saUspA*, cloned into pSVA407 with *Nco*I, *BamH*I	This study
pSVAaraFX-HA	pRN-1 based shuttle vector with *lacSSSO* reporter gene	(Van Der Kolk et al., 2020)
pSVA5132	Complementation of Δ*saUspA* with *saUspA* using its native promoter, cloned into pSVAaraFX-HA instead of *lacS* with *Sac*II and *Xho*I	This study
pSVA5133	Complementation of Δ*saUspA* with $saUspA^{\Delta dim}$ using its native promoter, cloned into pSVAaraFX-HA instead of *lacS* with *Sac*II and *Xho*I	This study

pSVA5140	Complementation of Δ*saUspA* with *saUspA*^{D8A} with its native promoter, cloned into pSVAaraFX-HA instead of *lacS* with *Sac*II and *Xho*I	This study
pSVA5141	Complementation of Δ*saUspA* with *saUspA*^{G97A} with its native promoter, cloned into pSVAaraFX-HA instead of *lacS* with *Sac*II and *Xho*I	This study
p7XNS3	Kan^{r}, expression plasmid containing replicon ColE1 (pBR322)	(Ye et al., 2020)
pSVA5134	*saUspA* cloned into p7XNS3 by FX cloning method	This study
pSVA5135	*saUspA*$^{\Delta dim}$ cloned into p7XNS3 by FX cloning method	This study
pSVA5138	*saUspA*^{D8A} cloned into p7XNS3 by FX cloning method	This study
pSVA5139	*saUspA*^{G97A} cloned into p7XNS3 by FX cloning method	This study

Table S2 Primers used in this study

primer	sequence (5´-3´)	purpose
	primers for pSVA5113	
7350	GAGGGATCCGACCCATTATCGACCTGAAG	Δ*saUspA* upstr fw
7351	TAATATGAAGGTACCTATACTCGTTGTAAAG	Δ*saUspA* upstr rev ol
7352	GTATAGGTACCTTCATATTAATATTTACTTAAGTGG	Δ*saUspA* downstr fw ol
7353	GAGCCATGGAGTCAGCCAATTCACTATCG	Δ*saUspA* down str rev
7362	GGCTGCAATGGAACCTAAAG	Δ*saUspA* check primer fw
7363	GGATATTTCTGATCCAATAT	Δ*saUspA* check primer rev
	primers for pSVA5132	
9119	GAGCTCGAGCTTTACAACGAGTATAGG	*saUspA* compl fw
9120	GAGCCGCGGCATTAGGCACCTATAGAATG	*saUspA* compl rev
	primers for pSVA5133	
9152	GAGCTCGAGCTTAGGTACCTTAGCCTTTTCTATTAAAGCG	$saUspA^{\Delta dim}$ compl fw
	primers for pSVA5134	
9166	ATATATGCTCTTCTAGTAAGTTTTTAATAGCGTATGATGG CTCA	*saUspA* expr. fw
9167	TATATAGCTCTTCATGCCTTTACAACGAGTATAGGTACCT TAGC	*saUspA* expr. rev
	Site-directed mutagenesis	
9179	GGCTCAGAACAGTCAAAAAAGGCTGTTAGATTTCTACTT CGC	$saUspA^{D8A}$ fw
9180	GCCTTTTTTGACTGTTCTGAGCCAGCATACGCTATTAAAA AC	$saUspA^{D8A}$ rev
9181	TAGTAGAGGTCTGACAGGAATTAAAAAAGTAGTAATAG GAAG	$saUspA^{G97A}$ fw
9182	ATTCCTGTCAGACCTCTACTAGCTGTAACTATCATATCAC AATTAAC	$saUspA^{G97A}$ rev
	primers for pSVA5135	
9151	TATATAGCTCTTCATGCCTTAGGTACCTTAGCCTTTTCTA TTAA	$saUspA^{\Delta dim}$ expr. rev
	Quantitative RT-PCR primers	
9123	AATAGCGTATGATGGCTCAG	*saUspA*-qRT-PCR-fw
9124	CTGACCACATCTCCACTTTC	*saUspA*-qRT-PCR-rev
2079	TAGCCAGGGTATGTTCAGTAATC	*upsA*-qRT-PCR-fw (Wolferen et al., 2015)
2080	ACCTAAGTTCCCGTTATTGAC	*upsA*-qRT-PCR-fw (Wolferen et al., 2015)
1480	CCTGCAACATCTATCCATAACATACCGA	*secY*-qRT-PCR-fw (Lassak et al., 2012)
1481	CCTCATAGTGTATATGCTTTAGTAGTAG	*secY*-qRT-PCR-rev (Lassak et al., 2012)

Supplementary References

Lassak, K., Neiner, T., Ghosh, A., Klingl, A., Wirth, R., and Albers, S. (2012). Molecular analysis of the crenarchaeal flagellum. 83, 110–124. doi:10.1111/j.1365-2958.2011.07916.x.

Le, T. N., Wagner, A., and Albers, S. (2017). A conserved hexanucleotide motif is important in UV-inducible promoters in Sulfolobus acidocaldarius Growth conditions. 778–788. doi:10.1099/mic.0.000455.

Li, L., Banerjee, A., Bischof, L. F., Maklad, H. R., Hoffmann, L., Henche, A., et al. (2017). Wing phosphorylation is a major functional determinant of the Lrs14-type biofilm and motility regulator AbfR1 in Sulfolobus acidocaldarius. 105, 777–793. doi:10.1111/mmi.13735.

Reimann, J., Lassak, K., Khadouma, S., Ettema, T. J. G., Yang, N., Driessen, A. J. M., et al. (2012). Regulation of archaella expression by the FHA and von Willebrand domain-containing proteins ArnA and ArnB in Sulfolobus acidocaldarius. 86, 24–36. doi:10.1111/j.1365-2958.2012.08186.x.

Van Der Kolk, N., Wagner, A., Wagner, M., Waßmer, B., Siebers, B., and Albers, S.-V. (2020). Identification of XylR, the activator of arabinose/xylose inducible regulon in Sulfolobus acidocaldarius and its application for homologous protein expression. *Front. Microbiol.* 11, 1066.

Wagner, M., Wolferen, M. Van, Wagner, A., Lassak, K., Meyer, B. H., Reimann, J., et al. (2012). Versatile genetic tool box for the crenarchaeote Sulfolobus acidocaldarius. 3, 1–12. doi:10.3389/fmicb.2012.00214.

Wolferen, M. Van, Ma, X., and Albers, S. (2015). DNA Processing Proteins Involved in the UV-Induced Stress Response of Sulfolobales. 197, 2941–2951. doi:10.1128/JB.00344-15.

Ye, X., Vogt, M. S., Van Der Does, C., BildlWolfgang, W., Schulte, U., Essen, L.-O., et al. (2020). The phosphatase PP2A interacts with ArnA and ArnB to regulate the oligomeric state and the stability of the ArnA/B complex. *Front. Microbiol.* 11, 1849. doi:10.3389/fmicb.2020.01849.
